# Suicide Behavior in Persons with Intellectual Disability

**DOI:** 10.1100/tsw.2005.91

**Published:** 2005-09-08

**Authors:** Joav Merrick, Efrat Merrick, Yona Lunsky, Isack Kandel

**Affiliations:** ^1^ National Institute of Child Health and Human Development, Ben Gurion University of the Negev, Beer-Sheva, Israel; ^2^ Division of Pediatrics, Ben Gurion University of the Negev, Beer-Sheva, Israel; ^3^ Center for Multidisciplinary Research in Aging, Faculty of Health sciences, Ben Gurion University of the Negev, Beer-Sheva, Israel; ^4^ Office of the Medical Director, Division for Mental Retardation, Ministry of Social Affairs, Jerusalem, Israel; ^5^ Centre for Addiction and Mental Health, University of Toronto, Canada; ^6^ Faculty of Social Science, Department of Behavioral Sciences, Academic College of Judea and Samaria, Ariel, Israel

**Keywords:** Suicide, intellectual disability, developmental disability, mental retardation, human development, holistic health, public health, Canada, Israel

## Abstract

Suicide is today in the Western world one of the leading causes of death and most people have had suicidal ideation at some time during their life. In the population of persons with intellectual disability some researchers have thought that impaired intellectual capacity could act as a buffer to suicidal behavior, but the fact is that the few studies conducted in that population contest this assumption and showed that the characteristics of suicidality in this population are very similar to persons without intellectual disability. This paper reviews the studies conducted and describe the symptomatology in this population. Professionals working with this population should therefore be aware of and assess for this behavior. Sadness or depression are symptoms that could indicate later suicidal behavior.

## INTRODUCTION

Suicide is today ranked as the 11th leading cause of death in the United States. Data from 2002 showed a rate of 11.0 per 100,000 population[1], and it is estimated that about one third of all people have suicidal ideation at some time during their lifetime[2]. With this in mind, it is interesting that so little attention in the scientific literature has been paid to suicidal behavior in persons with intellectual disability (ID). Both children, adolescents and adults with ID, mental retardation (MR), or developmental disabilities (DD) are at high risk for developing mental health problems. With a prevalence of psychopathology approximately four times higher than that found in the general population[3,4,5], there is still a tendency to under diagnose psychiatric disorders in this population. This is due to diagnostic overshadowing, lack of appropriate diagnostic criteria and appropriate assessment measures. People with ID also have a higher incidence of depression[6,7], and it is therefore interesting that the issue of suicide, in this population, has received very little interest by researchers[7]. Prevalence rates of suicide and suicide attempts in this population seems much lower, but it does occur[6,7].

In this paper, we review research on suicidal behavior in the population of persons with intellectual disability.

## SUICIDAL BEHAVIOR IN CHILDREN AND ADOLESCENTS

If there is very little research on suicide in adults with intellectual disability (ID)[7], then there is only a handful of papers concerning suicide in children and adolescents with ID, which are for the most part case reports.

Sternlicht et al[8] reviewed the charts for all residents of a state school for persons with ID and found 12 adolescents (mean IQ 63, range 48-79) who had attempted suicide, or revealed suicidal ideation. Kaminer et al[9] reported on three adolescents with mild/moderate ID with suicidal ideation, including one 16 year old with mild ID and schizoaffective disorder, who threatened to kill himself, since he was seeing and hearing scary things. They also speculated that maybe intellectual disability and its intellectual and adaptive limitations could work as a “buffer” against suicidal behavior, because of the lack of cognitive sophistication to conceptualize, plan or carry out suicide. If this holds true, then this could be the reason for the lack of papers on this topic with this population including children, adolescents and adults.

Menolascino et al[10] described eight persons with suicidal behavior out of 305 persons referred to an in-patient psychiatric facility where one 19 year old with mild ID was referred after his parents found him in his apartment with a knife and a suicide note.

A 35 year follow-up study on suicide mortality in a Finnish national cohort of 2,369 persons with ID[11] found ten cases of suicide and eight cases of undetermined external causes (UEC) of death, but none were below the age of 25 years.

Apart from the above case reports, we have only found two other studies, which we will describe in more details.

## RHODE ISLAND STUDY

Walters et al[12], from the Department of Psychiatry and Human Behavior at the Brown University School of Medicine in Rhode Island, studied 90 consecutive admissions to their dual diagnosis specialty unit at the children’s psychiatric hospital. Nineteen adolescents (10 males), with a mean age of 15.75 years and a mean IQ of 59 (range 37-86), were identified as suicidal (21% of the sample). As part of the extensive assessment, coding and observation during the hospitalizations (mean length 11.3 months), descriptions of suicidality were classified as:

- Ideation as verbal statements about death, dying or killing oneself, but without expressed intent to do so (like: “I do not want to be in this world anymore. I want to be dead.”).
- Threat as verbal statements about intent to hurt or kill oneself with no associated behavior (like: “I am going to choke myself until I die.”).
- Behavior as potentially harmful actions with/without verbal statements (like running from the unit towards a busy street after saying that he/she want to be dead).

A large proportion (79%) of these 19 adolescents had prior psychiatric hospitalizations (mean of 2.4) and their psychopathology could be classified into three categories: behavioral disorders (six cases), affective disorders (five cases), psychotic disorders (one case) and family conflicts (seven cases). In fact, 10% of the adolescents had been physically abused, 10% sexually abused and 26% both physical and sexually abused. Thirty-two percent had a history of suicidality prior to, or at the time of admission, 26% were suicidal only during hospitalization and 42% were suicidal both prior to and during hospitalization.

Out of the 19 adolescents, six were suicidal prior to admission and all expressed suicidal ideation, four made suicidal threats (66.6%) and three demonstrated suicidal behavior (50%). Of the other 13 who were suicidal only during hospitalization, or both prior to and during hospitalization, twelve (92.3%) expressed ideation, eleven (84.6%) made suicidal threats and nine (69.2%) demonstrated suicidal behavior.

This sample of 19 adolescents with mild-moderate intellectual disability showed suicidality characteristics similar to adolescents without intellectual disability, which disputes the earlier assumptions[9] of intellectual disability serving as a “buffer” against suicidal behavior in this population. There was a high prevalence of physical or sexual abuse of the adolescent with intellectual disability prior to hospitalization, which could be the trigger of suicidal behavior also seen in the general population. Another earlier study[13] found that 39% of multihandicapped children admitted to a psychiatric hospital were reportedly abused prior to the hospitalization.

## PITTSBURGH MEDICAL CENTER STUDY

Hardan and Sahl[14], from Western Psychiatric Institute and Clinic at University of Pittsburgh Medical Center, conducted a retrospective study of 233 patients over a 12 month period in their special program for children and adolescents with developmental and comorbid psychiatric disorders (after school partial hospitalization program, summer intensive treatment program, school based partial program, a 24 bed inpatient unit and an out-patient clinic).

They found that 47 (20%) (34 males, 13 females, mean age 10, range 4-18 years) had a past or present history of suicide ideation or attempt (SI/SA). Of the 47, there were 12 (25.5%) with borderline, 17 (36%) with mild and 5 (11%) with moderate intellectual disability. There were 22 in the total sample of 233 with severe/profound intellectual disability, but none had SI/SA.

Most of the 47 (94%) had had either SI only (13%), or in combination with threats and/or attempts, but only in four cases it could be said that the patient had an understanding of the concept of death and only one had a clear comprehension. Their psychopathologies were mostly behavioral disorders (28 cases), affective disorders (16 cases), psychotic disorders (one case) and family conflicts (17 cases). In this study, there is no mention of past or present physical or sexual abuse, but that does not mean that it did not take place.

Eight adolescents (17%) had suicidal ideation on admission, or experienced ideation during hospitalization, and in 23 cases (49%) an acute psychosocial stressor was associated with SI/SA (there is no mention of what this psychosocial stressor was, but could very well be abuse as found in other studies[12,13]).

The most observed behaviors in the suicidal group were impulsivity, poor concentration, hyperactivity, sadness, aggression and sleep disturbances. The three most often observed symptoms were sadness, somatization and eating disturbances.

## SUICIDAL BEHAVIOR IN ADULTS WITH INTELLECTUAL DISABILITY

Suicide in adults with intellectual disability (ID) has been reported [6,10,15,16,17,18], but very few in-depths studies have been conducted. One paper, from the Department of Psychiatry at Tufts University School of Medicine[6], in a retrospective study of the first psychiatric diagnostic evaluation for their most recent 100 adults with mild ID, 100 patients with moderate, severe or profound ID compared with 100 matching patients without ID (N-ID), showed that N-ID were significantly more likely to present with mood complaints, anxiety complaints and suicidality (14 patients). When mild ID was compared with moderate, severe and profound ID, it was found that mild ID was significantly more likely to present with anxiety complaints and suicidality (6 cases with mild ID and none with moderate, severe or profound ID).

A recent study, from the University of Toronto[7], included 98 adults with borderline to moderate ID from several community and one out-patient clinical setting providing service to this population, showed that 26 reported that they thought that life was not worth living "sometimes" (three with borderline, 18 with mild and five with moderate ID), while seven reported that they think this "most of the time" (six with mild and one with moderate ID). Of this total of 33 persons (34% of the sample), 23 said that they think about killing themselves and 11 said that they know how they will do it (three with an overdose of pills, three slashing wrists, four with a jump, one with knife and one shooting). A total of eleven reported earlier suicide attempts. An interesting finding was that 16 of the persons who had self-reported feeling suicidal were not rated as so by the informants. Meaning that, for 23% of the cases, family/staff were unaware of the risks.

When the different groups were compared, the suicidal adults were more likely to be unemployed, under greater stress, lonely, depressed, and have dual diagnosis and increased anxiety. They also reported less family support, less reciprocity in relationships and less overall social support. A review of the clinical charts revealed that the death of a relative or abuse history were common precursors to suicidal behavior

## DOWN SYNDROME AND SUICIDAL BEHAVIOR

A study, from Brown University School of Medicine[19], of 164 adults with Down syndrome found nine cases of depression with one who had suicidal ideation. This was a 23 year old female with moderate intellectual disability (ID) who lived with her recently divorced, depressed and hostile mother.

Another study[17] was of two case reports of suicide attempts which both occurred during major depressive periods. One was a 26 year old male who from adolescence had approached females without disabilities for dates, but was mostly rejected. With these rejections, he started suicidal ideation, burning himself with a lighter and finally jumped form a second story building, but was only slightly injured. The second case was a 25 year old female who ran away from home in a depressive state and attempted to throw herself in front of a car which missed her.

## SUICIDE MORTALITY IN PERSONS WITH INTELLECTUAL DISABILITY

One study, from the United Kingdom[20], of 204 sudden deaths in residential care over a 50 year period found one case of suicide from jumping from a bridge. Another study of mortality over 60 years, from a large US residential care center in California (21), did not report any case of suicide.

A 35 year follow-up study from Finland[11] was based on a national cohort of 2,369 people with ID reported 10 cases of suicide with most of them (six) residing in mental hospitals. Social support was lacking in all cases, and there was one case of sexual abuse. The overall suicide rate was 16.2 per 100,000 persons in this population, which was less than one third of the rate in the general Finish population.

A study of mortality and morbidity among older adults with ID, from the 1984-1993 period in New York (2,752 deaths of adults 40 years and older), showed a 9.5 per 100,000 rate for accidents, suicides and homicides[22].

In Israel, the Division for Mental Retardation (DMR) of the Ministry of Social Affairs provides service to about 25,000 persons with intellectual disability[23]. About 6,500 are provided service in residential care centers, about 2,000 in community living (hostels, protected apartments), while the rest are provided service, but live with their family[23].

The Office of the Medical Director has every case of death in residential, or community care, reported in order to review all cases and decide whether further investigation is warranted[24]. For the 1991-2005 period there have been no cases of suicide in this population[24,25].

## DISCUSSION

Suicide and suicide attempts in persons with intellectual disability (ID) is a topic that has barely been studied by professionals working with this population and, therefore, thought to be a rare phenomenon. The two studies mentioned above[12,14], from two psychiatric settings in the United States catering to the population of children/adolescents with developmental and intellectual disability from the 1995-1999 period, showed a frequency of 20-21% with suicidal behavior. These studies also showed that this behavior was more frequent in the in-patient setting, as a consequence of the worst cases being hospitalized.

In both studies[12,14], the characteristics, sex distribution and methods of suicide ideation and attempts were similar to that of adolescents without intellectual disability, but both studies had no case of completed suicide. The first study[12] had a high number of adolescents who were abused prior to admission, while the second study did not report on abuse, but this information (as in the general adolescent population) should alert every professional to investigate every case of attempted suicide in an adolescent for possible prior abuse (physical or sexual).

In adults with ID, from a community and out-patient sample in Canada, 34% reported that “life was not worth living” sometimes, 23% were thinking about killing themselves and 11% reported that they had attempted to kill themselves in the past[7], while an out-patient psychiatric sample from the US[6] reported a finding of 6%. It seems that suicidal behavior is common in persons with mild-moderate ID, while extremely rare in persons with severe-profound ID[6,7].

Most of the studies described a life event (family member death, abuse, rejection), dual diagnosis or depression as important risk factors. It therefore seems important to be able to diagnose depression in this population, but that is a problematic topic[26]. For those with mild-moderate ID there is a consensus to use standard diagnostic criteria, but for persons with severe-profound ID there is still doubt concerning the method of diagnosis[26].

## CONCLUSIONS

The clinical implication of the studies reviewed are that suicidal ideation and attempts do occur in the population of persons with intellectual disability and professionals should therefore be aware of and assess for this behavior. Sadness or depression are symptoms that could indicate later suicidal behavior.

## BIOSKETCHES

**Joav Merrick, MD, DMSc,** is professor of child health and human development affiliated with the Center for Multidisciplinary Research in Aging, Zusman Child Development Center, Division of Pediatrics and Community Health at the Ben Gurion University, Beer-Sheva, Israel, the medical director of the Division for Mental Retardation, Ministry of Social Affairs, Jerusalem, the founder and director of the National Institute of Child Health and Human Development. Has written numerous publications in the field of child health and human development, rehabilitation, intellectual disability, disability, health, welfare, abuse, advocacy, quality of life and prevention. Received the Peter Sabroe Child Award for outstanding work on behalf of Danish Children in 1985 and the International LEGO-Prize ("The Children's Nobel Prize") for an extraordinary contribution towards improvement in child welfare and well-being in 1987. E-Mail: jmerrick@internet-zahav.net. Website: www.nichd-israel.com

**Efrat Merrick, BSc** is a medical student at the Sackler School of Medicine, Tel Aviv University and a research assistant at the National Institute of Child Health and Human Development, Faculty of Health Sciences, Ben Gurion University of the Negev, Beer-Sheva, Israel. E-mail: efratmerrick@gmail.com

**Yona Lunsky, PhD, CPsych**, completed her PhD in Clinical Psychology at Ohio State University and the Nisonger Center, specializing in the area of dual diagnosis, followed by a two-year postdoctoral fellowship in dual diagnosis jointly through the University of Toronto Department of Psychiatry, the University of Guelph Department of Psychology and the Surrey Place Centre. Presently assistant professor at the University of Toronto in the Department of Psychiatry and a psychologist in the Dual Diagnosis Program at the Centre for Addiction and Mental Health, Canada. She has taught seminars on developmental disabilities to graduate level students in psychology and genetics, medical residents in psychiatry and family medicine, and university undergraduates and has provided psychological assessment and treatment to adults with a dual diagnosis in inpatient and outpatient settings. Her research interests include: risk factors for dual diagnosis, sexuality and developmental disability and women's health. E-mail: Yona_Lunsky@camh.net

**Isack Kandel, MA, PhD**, is senior lecturer at the Faculty of Social Sciences, Department of Behavioral Sciences, the Academic College of Judea and Samaria, Ariel. During the period 1985-93 he served as the director of the Division for Mental Retardation, Ministry of Social Affairs, Jerusalem, Israel. E-mail: kandelii@zahav.net.il
